# Supplementary material for: Feline Infectious Peritonitis Effusion Index: A Novel Diagnostic Method and Validation of Flow Cytometry-Based Delta Total Nucleated Cells Analysis on the Sysmex XN-1000V®
Source: Vet Sci. 2024 Nov 13;11(11):563. doi: 10.3390/vetsci11110563 (PMC11598915; doi:10.3390/vetsci11110563)
Supplement: Supplementary file 1 [file vetsci-11-00563-s001.zip › vetsci-3247418-supplementary.pdf]

Table S1. Spearman correlation analysis: biochemical and flow cytometry data in effusions, categorised according to the feline coronavirus RT-qPCR test

		Age (Years)	Specific Gravity (SG)	TP (g/dL)	ALB (g/dL)	GLOB (g/dL)	ALB/GLOB ratio	WBC–BF (10^3/μL)	TC–BF (10^3/μL)	TNCC–WDF (10^3/μL)	TNCC–WNR (10^3/μL)	ΔTCN	FCoV RT–qPCR	FIP Effusion Index
Age (Years)	*r*	1	0.11	0.02	0.08	-0.01	0.05	0.01	0.01	0.08	0.07	-0.05	0.01	-0.07
	p		0.507	0.877	0.626	0.937	0.749	0.94	0.934	0.619	0.674	0.735	0.929	0.659
Specific Gravity (SG)	*r*	0.11	1	0.89	0.07	0.81	-0.61	0.27	0.27	0.16	-0.41	0.55	0.6	0.55
	p	0.507		<.001	0.671	<.001	<.001	0.083	0.078	0.299	0.007	<.001	<.001	<.001
TP (g/dL)	*r*	0.02	0.89	1	-0.18	0.96	-0.83	0.4	0.4	0.22	-0.5	0.67	0.78	0.71
	p	0.877	<.001		0.25	<.001	<.001	0.009	0.008	0.165	0.001	<.001	<.001	<.001
ALB (g/dL)	*r*	0.08	0.07	-0.18	1	-0.38	0.65	-0.1	-0.1	-0.04	0.32	-0.38	-0.49	-0.47
	p	0.626	0.671	0.25		0.013	<.001	0.539	0.522	0.81	0.041	0.014	0.001	0.002
GLOB (g/dL)	*r*	-0.01	0.81	0.96	-0.38	1	-0.93	0.39	0.4	0.2	-0.51	0.67	0.81	0.75
	p	0.937	<.001	<.001	0.013		<.001	0.01	0.009	0.2	0.001	<.001	<.001	<.001
ALB/GLOB ratio	*r*	0.05	-0.61	-0.83	0.65	-0.93	1	-0.37	-0.38	-0.2	0.48	-0.65	-0.79	-0.75
	p	0.749	<.001	<.001	<.001	<.001		0.015	0.013	0.201	0.001	<.001	<.001	<.001
WBC–BF (10^3/μL)	*r*	0.01	0.27	0.4	-0.1	0.39	-0.37	1	1	0.52	0.03	0.24	0.31	0.27
	p	0.94	0.083	0.009	0.539	0.01	0.015		<.001	<.001	0.84	0.133	0.046	0.088
TC–BF (10^3/μL)	*r*	0.01	0.27	0.4	-0.1	0.4	-0.38	1	1	0.51	0.03	0.24	0.32	0.27
	p	0.934	0.078	0.008	0.522	0.009	0.013	<.001		0.001	0.848	0.133	0.038	0.086
TNCC–WDF (10^3/μL)	*r*	0.08	0.16	0.22	-0.04	0.2	-0.2	0.52	0.51	1	0.44	0.13	0.12	0.12
	p	0.619	0.299	0.165	0.81	0.2	0.201	<.001	0.001		0.003	0.421	0.453	0.433
TNCC–WNR (10^3/μL)	*r*	0.07	-0.41	-0.5	0.32	-0.51	0.48	0.03	0.03	0.44	1	-0.81	-0.72	-0.8
	p	0.674	0.007	0.001	0.041	0.001	0.001	0.84	0.848	0.003		<.001	<.001	<.001
ΔTCN	*r*	-0.05	0.55	0.67	-0.38	0.67	-0.65	0.24	0.24	0.13	-0.81	1	0.85	0.97
	p	0.735	<.001	<.001	0.014	<.001	<.001	0.133	0.133	0.421	<.001		<.001	<.001
FCoV RT–qPCR	*r*	0.01	0.6	0.78	-0.49	0.81	-0.79	0.31	0.32	0.12	-0.72	0.85	1	0.86
	p	0.929	<.001	<.001	0.001	<.001	<.001	0.046	0.038	0.453	<.001	<.001		<.001
FIP Effusion Index	*r*	-0.07	0.55	0.71	-0.47	0.75	-0.75	0.27	0.27	0.12	-0.8	0.97	0.86	1
	p	0.659	<.001	<.001	0.002	<.001	<.001	0.088	0.086	0.433	<.001	<.001	<.001	

r, correlation; *p* significance; TP (g/dL), absolute levels of total proteins; ALB (g/dL), absolute levels of albumin; GLOB (g/dL), absolute levels of globulins; WBC-BF (10^3/μL), total white blood cells count in body fluid mode; TC-BF (10^3/μL), total nucleated cells in body fluid mode; TNCC-WDF (10^3/μL), total nucleated cells in the white blood cell differential channel in whole blood mode; TNCC-WNR (10^3/μL), total nucleated cells in the white blood cell nucleated channel in whole blood mode; ΔTNC, total nucleated cell derived from the ratio between TNCC-WDF and TNCC-WNR counts on the Sysmex XN-1000V®; FCoV RT-qPCR, feline coronavirus real-time quantitative reverse transcription polymerase chain reaction test result; FIP Effusion Index, feline infectious peritonitis effusion index (calculated by dividing the delta total nucleated cell (ΔTNC) count by the albumin-to-globulin ratio (ALB/GLOB ratio).
